# Application of an Autophagy-Related Gene Prognostic Risk Model Based on TCGA Database in Cervical Cancer

**DOI:** 10.3389/fgene.2020.616998

**Published:** 2021-02-09

**Authors:** Huadi Shi, Fulan Zhong, Xiaoqiong Yi, Zhenyi Shi, Feiyan Ou, Zumin Xu, Yufang Zuo

**Affiliations:** Cancer Center, Affiliated Hospital of Guangdong Medical University, Zhanjiang, China

**Keywords:** cervical cancer, prognosis, nomogram, autophagy, TCGA

## Abstract

**Background:** Autophagy plays an important role in the development of cancer. However, the prognostic value of autophagy-related genes (ARGs) in cervical cancer (CC) is unclear. The purpose of this study is to construct a survival model for predicting the prognosis of CC patients based on ARG signature.

**Methods:** ARGs were obtained from the Human Autophagy Database and Molecular Signatures Database. The expression profiles of ARGs and clinical data were downloaded from the TCGA database. Differential expression analysis of CC tissues and normal tissues was performed using R software to screen out ARGs with an aberrant expression. Univariate Cox, Lasso, and multivariate Cox regression analyses were used to construct a prognostic model which was validated by using the test set and the entire set. We also performed an independent prognostic analysis of risk score and some clinicopathological factors of CC. Finally, a clinical practical nomogram was established to predict individual survival probability.

**Results:** Compared with normal tissues, there were 63 ARGs with an aberrant expression in CC tissues. A risk model based on 3 ARGs was finally obtained by Lasso and Cox regression analysis. Patients with high risk had significantly shorter overall survival (OS) than low-risk patients in both train set and validation set. The ROC curve validated its good performance in survival prediction, suggesting that this model has a certain extent sensitivity and specificity. Multivariate Cox analysis showed that the risk score was an independent prognostic factor. Finally, we mapped a nomogram to predict 1-, 3-, and 5-year survival for CC patients. The calibration curves indicated that the model was reliable.

**Conclusion:** A risk prediction model based on CHMP4C, FOXO1, and RRAGB was successfully constructed, which could effectively predict the prognosis of CC patients. This model can provide a reference for CC patients to make precise treatment strategy.

## Introduction

Autophagy is a self-degradative process that is important for maintaining nutrient and energy homeostasis and eliminating intracellular pathogens (Glick et al., 2010; Jiang and Mizushima, 2014; Yu et al., 2018a). Autophagy is generally considered to be a survival mechanism and widely involved in a variety of pathophysiological processes such as cancer, metabolism, and cardiovascular disease (Choi et al., 2013). Cancer cells have evolved to use autophagy as an adaptive mechanism to survive under extreme stress in the tumor microenvironment and to enhance the resistance of anticancer drugs. Regulation of autophagy has become a promising cancer treatment strategy (Pietrocola et al., 2016; Chude and Amaravadi, 2017; Marinkovic et al., 2018). However, autophagy plays a dual role in many cancers both promoting and suppressing cancers, depending on the tumor microenvironment (Yun and Lee, 2018). It is of great significance to further explore the potential role of autophagy in tumor genesis and development.

Cervical cancer (CC) is a common gynecological malignant tumor worldwide. Statistics showed that about 311,000 people died of CC globally in 2018 (Bray et al., 2018). The incidence of CC has declined in recent years as screening and increasing health literacy. However, CC remains the leading cause of cancer-related deaths among women in developing countries (Arbyn et al., 2020). The main treatment methods for CC patients are radical hysterectomy and radical radiotherapy plus concurrent cisplatin chemotherapy. Nevertheless, some patients still relapse after surgery or radiotherapy. Patients with relapse have limited treatment and a poor prognosis (Dizon et al., 2014). To further improve effectiveness of treatment and develop precise treatment strategies, oncologists need to identify the prognosis of CC patients. Therefore, it is important to explore new biomarkers suitable for CC prognosis prediction.

In recent years, the prognosis risk model based on ARG expression signature has been applied in lung cancer (Liu et al., 2019), gastric cancer (Qiu et al., 2020), etc. To our knowledge, the prognostic role of ARGs in CC is unclear. In this study, we performed a biological information comprehensive analysis on the transcriptome and clinical information of the CESC cohort from the TCGA database. The differentially expressed ARGs were screened out, then the prognostic model based on ARGs was constructed by multivariate Cox regression. Meanwhile, we mapped a nomogram which might provide a new reference index for the stratification of prognosis risk and treatment strategy selection of CC patients.

## Methods

### Acquisition of Human Autophagy-Related Gene Sets

Human Autophagy Database (HADb, http://autophagy.lu/clustering/index.html) is a public database containing information of ARGs (Moussay et al., 2011). We obtained 232 ARGs from HADb. Meanwhile, we also obtained 394 ARGs from the GO_AUTOPHAGY gene set in the Molecular Signatures Database (Wang et al., 2020) (MSigDB v6.2, http://software.broadinstitute.org/gsea/msigdb). The overlapping ARGs of the two databases were deleted. Finally, we obtained 531 ARGs.

### Gene Expression Information and Clinical Data of Cervical Cancer

Information of gene expression and clinical data was downloaded from the level-3 gene-expression information (FPKM normalized) of the TCGA-CESC cohort (https://portal.gdc.cancer.gov/). Perl 5.28.1 software was used to merge the original data and extract the expression data of all ARGs. The collected clinicopathological data included age, stage, grade, survival status, and survival duration in days. Our research excluded any samples that had missing or insufficient data on age, grade, stage, survival status, and survival duration. We retained both RNA-Seq and clinical data, which we used for further investigation. Our study was in accordance with the publication guidelines provided by TCGA.

### Identification of Differential Expression ARGs and Enrichment Analysis

The “edgeR” package of R 3.6.1 software was used to analyze the differential expression of ARGs (DE-ARGs) in 306 cervical cancer tissues and 3 normal tissues. The screening criteria are as follows: false discovery rate (FDR) <0.05, fold change ≥2. Gene ontology (GO) functional enrichment (including biological process, cell components, and molecular functions) and Kyoto Encyclopedia of Genes and Genomics (KEGG) signaling pathways were analyzed and visualized by “clusterprofiler,” “org.HS.eg.db,” “enrichplot,” and “ggplot2” package of R 3.6.1 software.

### Identification of Prognostic Gene Signatures

The entire set was randomly divided into train set and test set according to the ratio of 6:4. Univariate Cox regression analysis was performed on DE-ARGs of the train set to eliminate the genes, which might not be related to the prognosis of CC patients. Hazard ratio (HR) and *P*-value of each DE-ARGs were calculated. When the *P* < 0.05, the gene was selected for further analysis. Lasso regression analysis was used to reduce the collinearity between genes and prevent overfitting of prognostic risk model variables. By constructing penalty function, the regression coefficient of independent variables was compressed to achieve the dimension reduction of gene data, and then DE-ARGs with a high correlation of prognosis were obtained. Subsequently, we performed further variable filtering through the step function of R language, which was a stepwise regression analysis based on AIC information statistics. The smallest AIC information statistics was selected to delete or increase variables. The mode of stepwise search includes “backward” and “forward.” Finally, multivariate Cox regression analysis was performed on DE-ARGs obtained by Lasso regression screening. The multivariate regression coefficient of each DE-ARGs was calculated to obtain the key DE-ARGs related to the prognosis of CC patients.

### Construction and Validation of the Prognostic Risk Score Model Based on DE-ARGs

According to the key DE-ARGs obtained by Cox and Lasso regression screening, the risk score equation was constructed (Tibshirani, 1997; Yu et al., 2018b): risk score = $\;\overset{n}{\underset{i=1}{\sum }}{\text{Coef}}_{\text{i}}$ × X_i_. Coef refers to the regression coefficient of DE-ARGs in multivariate Cox regression analysis; X is the expression value of the gene; and n is the number of prognostic DE-ARGs. Patients were divided into low-risk and high-risk groups according to the median of risk score as cutoff value. The “survival” package of R software was used for Kaplan–Meier survival analysis. The ROC curve of the model was drawn using the “timeROC” package of R software to evaluate the sensitivity and specificity of the prognostic model. Principal component analysis was performed to explore the distribution pattern of high- and low-risk groups according to ARG gene expression. To investigate whether risk score could be an independent predictor of overall survival (OS) in CC patients, univariate and multivariate Cox regression analyses were performed. Age, grade, clinical stage, and risk score were used as covariables. The test set and the entire set were used to validate the prognostic risk score model based on DE-ARGs. In addition, GSE44001 containing disease-free survival (DFS) and mRNA expression data was downloaded from the GEO database as a validation set. The risk score for each patient was calculated with the same formula as a training set. The Kaplan–Meier curve was used to assess the predictive ability of the autophagy-related signature.

### The Construction of Nomogram and Calibration Curves

A clinical practical nomogram was established to predict individual survival probability by the “rms” package of R software. To assess the consistency between actual and predicted survival in the nomogram, calibration curves for predicting 1-, 3-, and 5-year survival rate were also drawn.

## Results

### Identification and Enrichment Analysis of DE-ARGs

**Figure 1:**
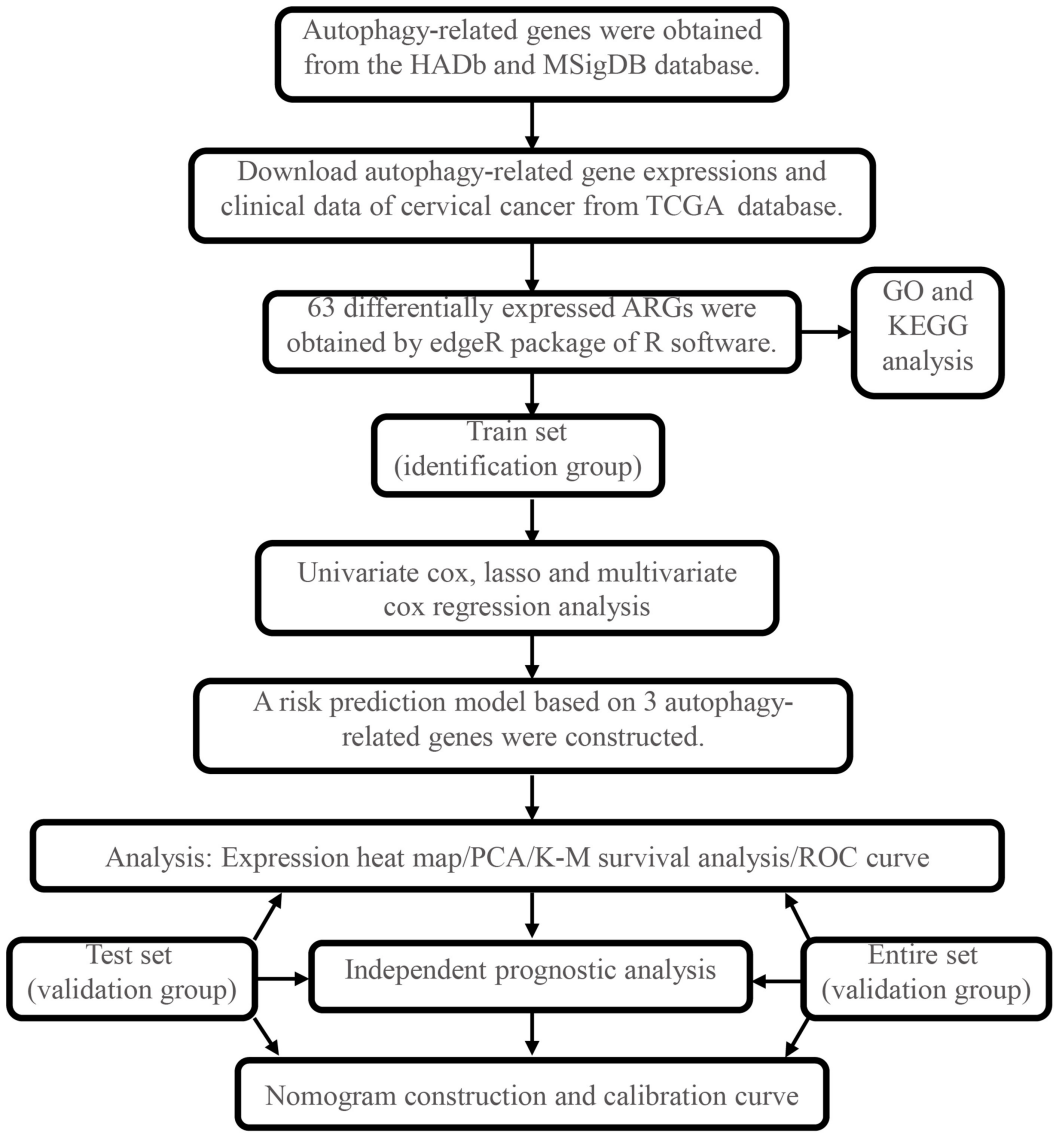
Flowchart for identifying the autophagy-related prognostic signature.

**Figure 2:**
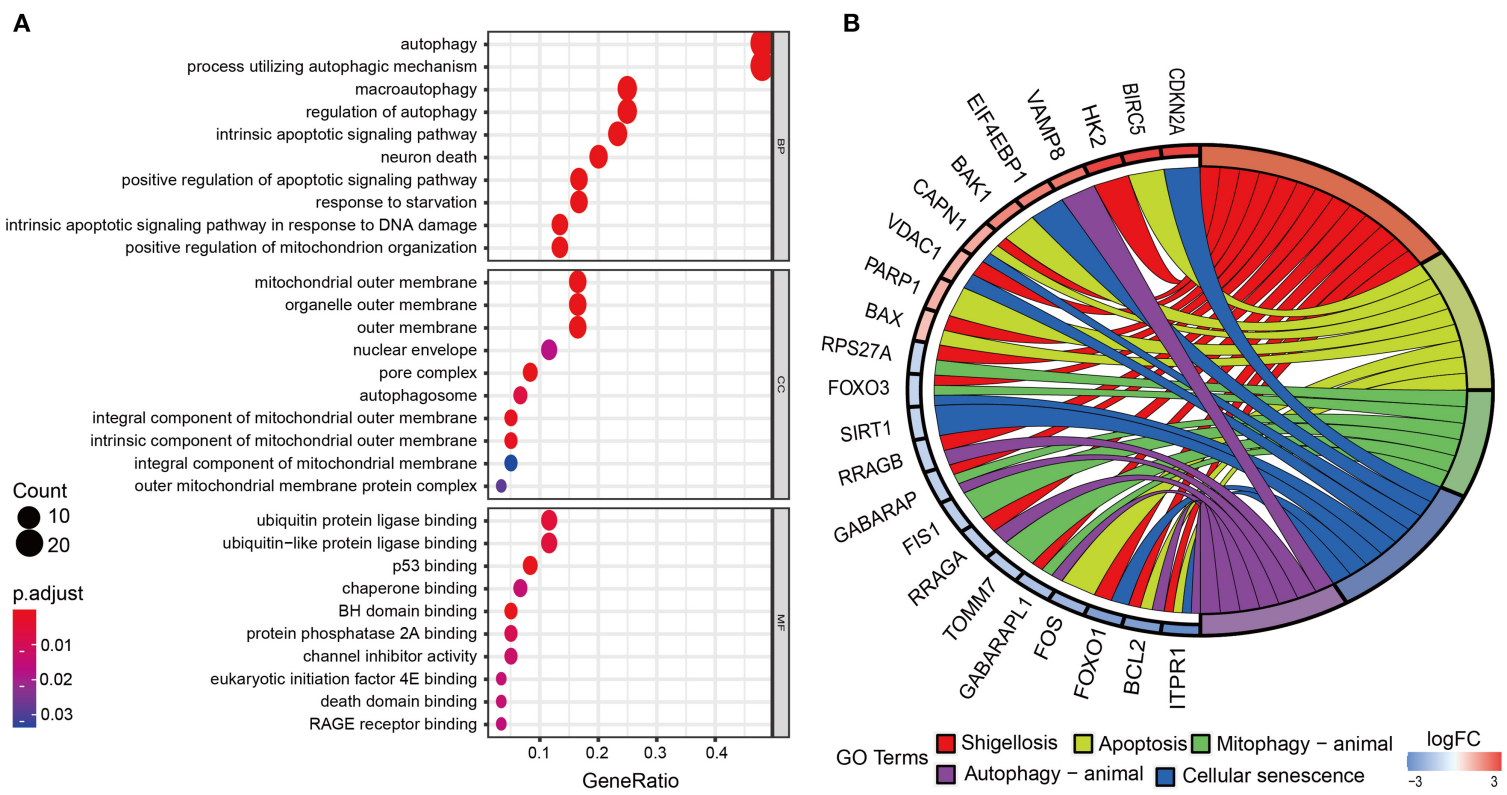
Gene ontology (GO) functional enrichment and Kyoto Encyclopedia of Genes and Genomes (KEGG) pathway analysis. **(A)** Enrichment analysis reveals the top 10 GO terms. **(B)** KEGG pathway analysis. According to the adjusting *P*-value, the node color increases from red to blue. The size of the node indicates the number of counts.

The overall process of this study is described in Figure 1. In this work, we collected mRNA expression profiles with 306 cervical cancer samples and 3 normal samples from the TCGA database. The heat map of differential expression of ARGs was drawn, as shown in Supplementary Figure 1A. Compared with normal cervical tissue, there were 63 ARGs with differential expression in cervical cancer tissue (Supplementary Figure 1B). The green dots represent 32 downregulated ARGs, and the red dots represent 31 upregulated ARGs in CC tissue. Figure 2A shows the GO functional enrichment analysis. In the aspect of biological processes, DE-ARGs were mostly enriched in autophagy, process utilizing autophagic mechanism and macro-autophagy, etc. In the aspect of cell component involving mitochondrial outer membrane, organelle outer and outer membrane, etc., and in the aspect of molecular function, DE-ARGs were mostly enriched in ubiquitin protein ligase binding, ubiquitin-like protein ligase binding, and p53 binding. Besides, KEGG signaling pathway analysis showed that DE-ARGS were involved in some cancer-related signaling pathway such as apoptosis, cellular senescence, p53 signaling pathway, and HIF-1 signaling pathway (Figure 2B).

### Construction of Prognostic Markers of CC in the Train Set

The DE-ARG expression profiles and clinical follow-up information of CC were merged to screen out 306 cervical cancer samples. The entire set was randomly divided into train set and test set with a ratio of 6 to 4. The train set and test set are 184 and 122 samples, respectively. Univariate Cox regression analysis was performed on 63 DE-ARG, and then Lasso regression analysis was conducted (Supplementary Figures 2A,B). Finally, a prognostic model using the expression of CHMP4C, FOXO1, and RRAGB was conducted by multivariate Cox regression. The coefficients of each gene are shown in Table 1.

**Table 1:** Three prognostic DE-ARGs was associated with OS.

		**Hazard ratio**	
**Gene name**	**Coefficient**	**(95% confidence interval)**	***P*-value**
FOXO1	0.113493	1.120 (1.02–1.22)	0.012
CHMP4C	0.044977	1.046 (1.00–1.10)	0.067
RRAGB	−0.163103	0.850 (0.73–0.99)	0.039

*ARGs, autophagy-related genes; OS, overall survival; DE, differential expression*.

### Correlation Between the Risk Score and OS in the Train Set

**Figure 3:**
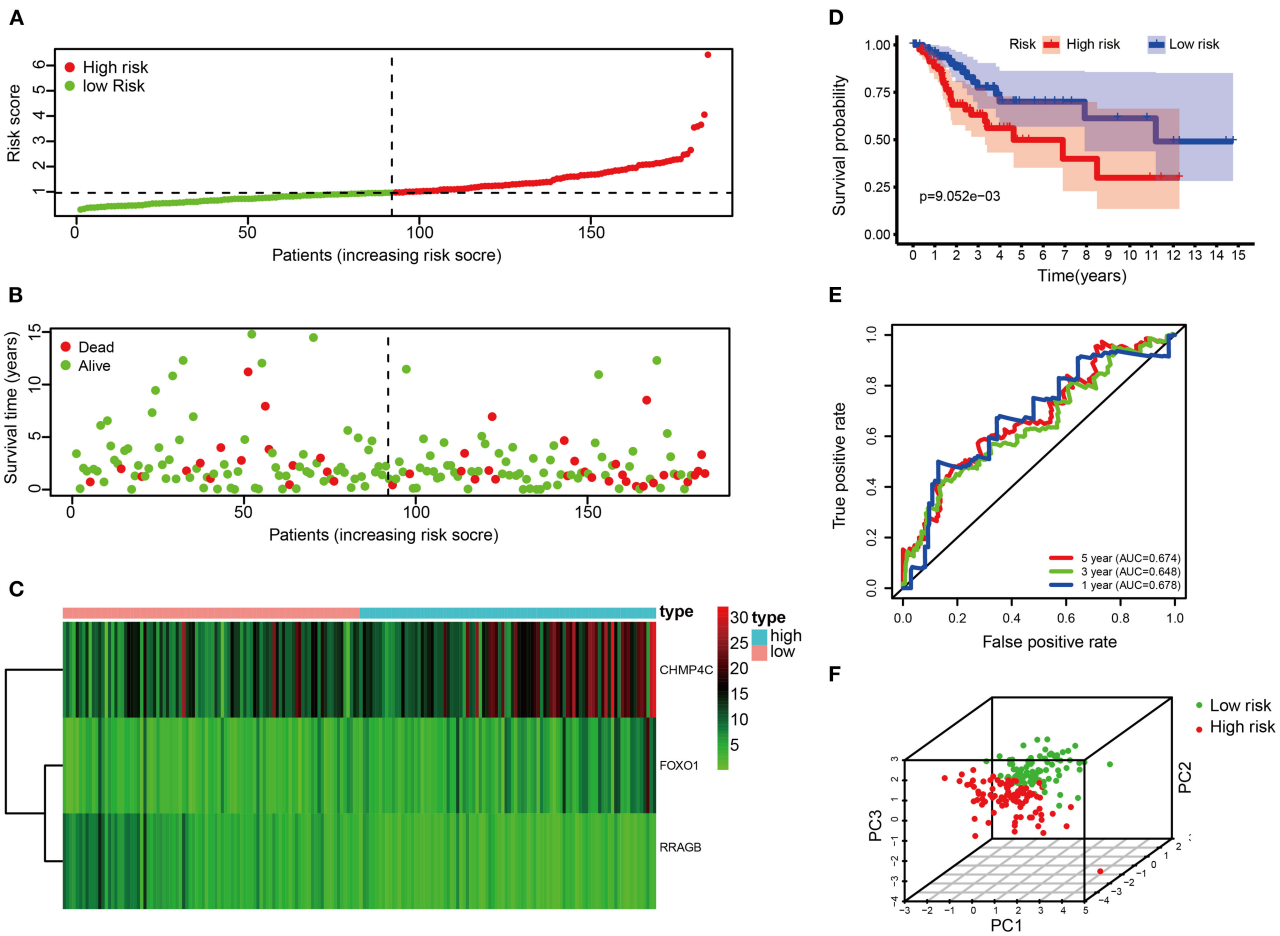
Correlation between the risk score and overall survival in the train set. **(A,B)** Distribution of risk score and patient survival status of cervical cancer. **(C)** Expression heat map of 3 DE-ARGs. **(D)** The Kaplan–Meier curve demonstrates that patients in the high-risk group have a poorer prognosis. **(E)** Time-dependent ROC curve analysis for survival prediction by the risk score. **(F)** Principal component analysis.

Each patient's risk score was calculated based on the prognostic model consisting of these three DE-ARGs. These include two potential risky genes and one potential protective gene. The risk score was quantified by the following formula: risk score = (0.113493 × FOXO1) + (0.044977 × CHMP4C) + (−0.163103 × RRAGB). Patients were divided into low-risk (*n* = 91) and high-risk groups (*n* = 91) according to the median of risk score. The distribution of risk score, survival status, the heat map of these 3 prognostic DE-ARGs in the train set are shown in Figures 3A–C. The Kaplan–Meier curve demonstrated that patients in the high-risk group have a poorer prognosis (*P* < 0.001, Figure 3D). Time-dependent ROC analysis is shown in Figure 3E. The values of the area under the receiver operating characteristic curve (AUC) were 0.678, 0.648, and 0.674, respectively, for predicting 1-, 3-, and 5-year survival rates. Principal component analysis showed that the distribution patterns of high-risk and low-risk populations were different based on the train set (Figure 3F).

### Validation of the Risk Score in the Test Set

**Figure 4:**
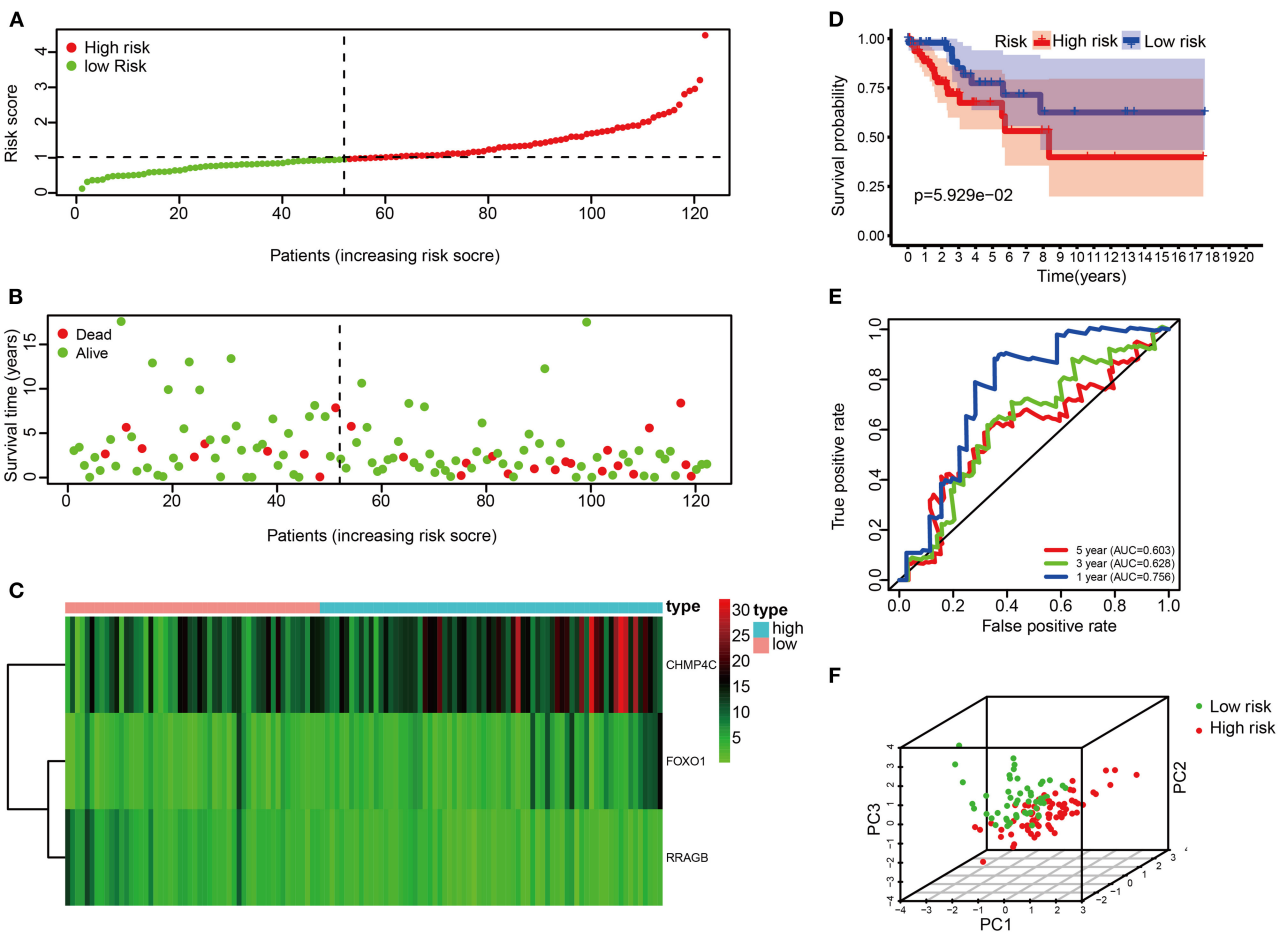
Correlation between the risk score and overall survival in the test set. **(A,B)** Distribution of risk score and patient survival status of cervical cancer. **(C)** Expression heat map of 3 DE-ARGs. **(D)** The Kaplan–Meier curve demonstrates that patients in the high-risk group have a poorer prognosis. **(E)** Time-dependent ROC curve analysis for survival prediction by the risk score. **(F)** Principal component analysis.

The risk score of each patient in the test set was calculated according to the same risk score formula of the train set. The patients of the test set were divided into the high-risk group (*n* = 70) and low-risk group (*n* = 52) based on the cutoff value of the train set. The distribution of risk score, the survival status, and the heat map of these 3 prognostic DE-ARGs in the test set are shown in Figures 4A–C. Consistent with the results of the train set, patients in the high-risk group had a poorer prognosis in test set (*p* < 0.001, Figure 4D). Moreover, the values of AUC were 0.756, 0.628, and 0.603, respectively, for predicting 1-, 3-, and 5-year survival rates (Figure 4E). Principal component analysis showed that the distribution patterns of high-risk and low-risk populations were different based on the train set (Figure 4F).

### Validation of the Risk Score in the Entire Set

**Figure 5:**
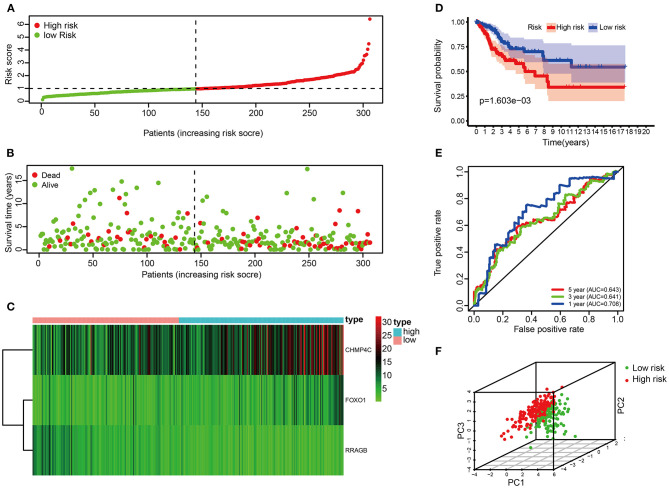
Correlation between the risk score and overall survival in the entire set. **(A,B)** Distribution of risk score and patient survival status of cervical cancer. **(C)** Expression heat map of 3 DE-ARGs. **(D)** The Kaplan–Meier curve demonstrates that patients in the high-risk group have a poorer prognosis. **(E)** Time-dependent ROC curve analysis for survival prediction by the risk score. **(F)** Principal component analysis.

Risk score of the entire set were used to further validate the prognostic model. The risk score of each patient in the entire set was calculated according to the same risk score formula of the train set. The patients of the entire set were divided into high-risk (*n* = 162) and low-risk groups (*n* = 144) based on the cutoff value of the train set. The distribution of risk score, the survival status, the heat map of these 3 prognostic DE-ARGs in the entire set are shown in Figures 5A–C. Consistent with the above findings, patients in the high-risk group have a poorer prognosis (*p* < 0.001, Figure 5D). In addition, the values of AUC were 0.708, 0.641, and 0.643, respectively, for predicting 1-, 3-, and 5-year survival rates (Figure 5E). Principal component analysis showed that the distribution patterns of high-risk and low-risk populations were different based on the train set (Figure 5F). To verify the inclusiveness of the model, cervical cancer patients were divided into different subsets based on FIGO stage and pathological grade. Consistent with the above findings, patients in the high-risk group have a poorer prognosis in different subsets of CC patients (stages I & II, stages III & IV, grades 1 & 2 and 3 & 4, *p* < 0.001, Supplementary Figures 3A–D). It is suggested that the model has a good inclusiveness.

### Validation of the Risk Score in an External Dataset

To validate the predictive ability of autophagy-related signature, risk scores were calculated with the same formula for patients in GSE44001. We extracted the disease-free survival (DFS) and expression data of the three ARGs from this dataset and calculated the risk score of each sample. Consistent with the results of the TCGA dataset, patients in the high-risk group had a lower DFS rate in this dataset (*p* < 0.001, Supplementary Figure 4A). In addition, the values of AUC were 0.539, 0.563, and 0.609, respectively, for predicting 1-, 3-, and 5-year disease-free survival rates (Supplementary Figure 4B).

### Autophagy as an Independent Prognostic Factor

**Figure 6:**
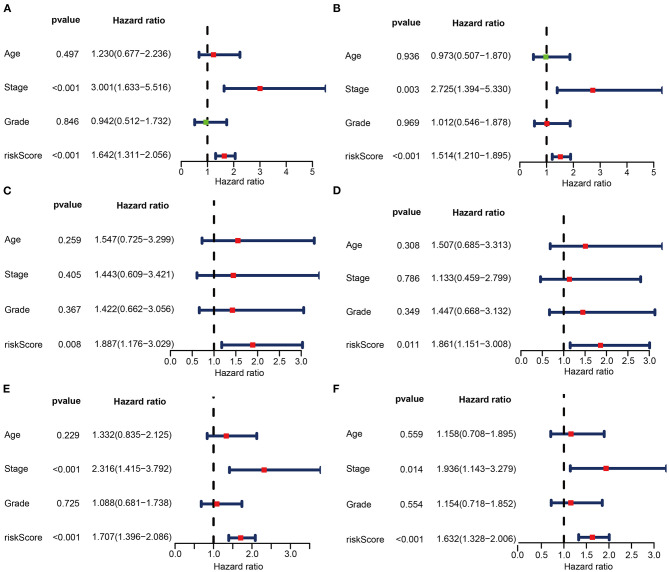
Univariate **(A,C,E)** and multivariate **(B,D,F)** regression analyses of the prognostic value for the train set **(A,B)**, the test set **(C,D)**, and the entire set **(E,F)** with clinicopathologic factors.

To investigate whether risk score could be an independent predictor of OS in CC patients, univariate and multivariate Cox regression analyses were performed. In the train set, risk score was significantly associated with OS in univariate Cox regression analysis (HR = 1.642, 95% CI = 1.311–2.056, *P* < 0.001, Figure 6A). Multivariate analysis showed that the risk score was an independent prognostic indicator (HR = 1.514, 95% CI = 1.210–1.895, *P* < 0.001, Figure 6B). Likewise, univariate Cox regression analysis showed that the risk score was associated with OS in the test set (HR = 1.887, 95% CI = 1.176–3.029, *P* = 0.008, Figure 6C). Multivariate analysis showed that the risk score was an independent prognostic indicator in the test set (HR = 1.861, 95% CI = 1.151–3.008, *P* = 0.011, Figure 6D). Consistent with the above findings, the risk score was significantly associated with OS in univariate Cox regression analysis in the entire set (HR = 1.707, 95% CI = 1.396–2.086, *P* < 0.001, Figure 6E). Multivariate analysis showed that the risk score was an independent prognostic indicator (HR = 1.632, 95% CI = 1.328–2.006, *P* < 0.001, Figure 6F).

### The Construction of Nomogram and Calibration Curves

**Figure 7:**
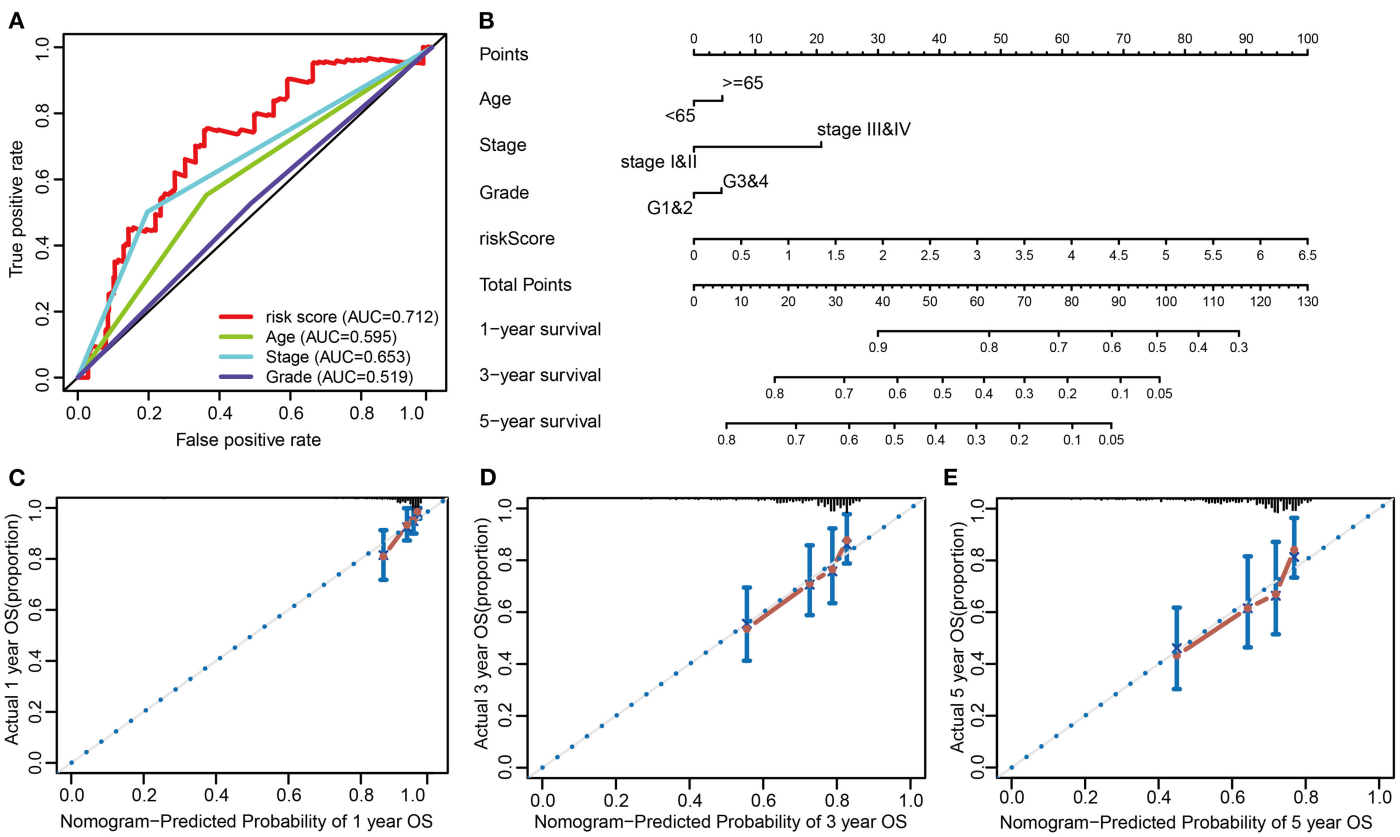
Nomogram to predict the probability of patients with cervical cancer. **(A)** ROC curve analysis. **(B)** The nomogram to predict 1-, 3-, or 5-year OS in the entire set. **(C–E)** The calibration plots for predicting patient 1-, 3-, or 5-year OS.

To provide a better quantitative method for clinicians to predict cancer prognosis, a nomogram was constructed by combining the risk score with other clinicopathological risk factors. The nomogram showed that our risk score was the most important factor among the various clinical parameters (Figure 7B). The predictive abilities of the nomogram were analyzed by the AUC values (AUC of 1-year OS = 0.712, Figure 7A). The model's AUC value (risk score, AUC value = 0.712) was higher than that of International Federation of Gynecology and Obstetrics (FIGO) staging (AUC value = 0.653). It is suggested that the model constructed on 3 ARGs has better accuracy and specificity than FIGO staging. In addition, calibration curves revealed that the predicted and actual survival rates were well matched at 1-, 3-, and 5 years (Figures 7C–E). These findings suggested that the nomogram has a high accuracy in predicting overall survival.

## Discussion

The current widely accepted method of CC staging is the International Federation of Gynecology and Obstetrics (FIGO) staging system (Oncology FCo, 2014). The 2018 FIGO staging system is mainly based on imaging or pathological examination. However, staging is based on imaging and subjective judgment of doctors' physical examination for some non-surgical patients (Oncology FCo, 2014). Therefore, if the patient is accompanied by pelvic inflammation, endometriosis, or obesity, this is inherently inaccurate. Due to the significant differences between clinically determined stages and surgical pathological results, the prognosis of patients with FIGO stages was significantly different (Li et al., 2016). With the rapid development of knowledge of cancer biology and the discovery and validation of biological factors that predict cancer outcome and treatment response, some oncologists are increasingly using a variety of related, non-anatomical (including molecular) factors to predict individual patient outcome (Salib et al., 2020).

Some studies have found that autophagy is involved in the development of CC. Zhang et al. showed that MAP7 promoted migration and invasion and progression of human cervical cancer through modulating the autophagy (Zhang et al., 2020). Wu et al. found that kindlin 2 suppressed cervical cancer cell migration through AKT/mTOR-mediated autophagy induction (Wu et al., 2020). Wang et al. showed that angelicin inhibited the malignant behavior of human cervical cancer potentially via inhibiting autophagy (Wang et al., 2019). In recent years, gene signatures have been used to predict the prognosis of various cancers. To some extent, it is even better than TNM staging and histopathological diagnosis (Karamichalis et al., 2016; Chlis et al., 2018). Prognostic models based on autophagy-related gene expression have been reported for various cancers, such as lung cancer (Liu et al., 2019) and gastric cancer (Qiu et al., 2020). To our knowledge, prognostic models based on ARGs and clinicopathological characteristics in CC have not been reported. In this study, ARGs with a differential expression were screened out from CC tissues and normal tissues. Subsequently, the prognostic model based on FOXO1, CHMP4C, and RRAGB was successfully constructed by Lasso and Cox regression. Patients in the high-risk group had a poor prognosis. The ROC curve showed that the predictive abilities of the nomogram were higher than those of the TNM staging system, which is consistent with previous studies (Li et al., 2018; Yu and Zhang, 2019). The calibration curves indicated that the predicted value of the nomogram had a high degree of coincidence with the true value. The nomogram is a valuable tool to predict the prognosis of individual patients. In recent years, the nomogram has become increasingly popular for its ability to construct statistical prognostic models using different variables (Balachandran et al., 2015; Jeong et al., 2020). Our model can provide a new reference for prognostic risk stratification assessment and treatment strategy selection in patients with cervical cancer.

In the present study, GO functional enrichment and KEGG signaling pathway analysis showed that DE-ARGS were involved in some cancer-related signaling pathway such as autophagy, apoptosis, p53 signaling pathway, and HIF-1 signaling pathway. In addition, we successfully constructed a prognostic model based on three DE-ARGs with a prognostic value. These three DE-ARGs have been reported to be associated with cancer, of which CHMP4C and FOXO1 have been shown to play an important role in the occurrence and development of cervical cancer (Xie and Xie, 2019; Lin et al., 2020; Yang et al., 2020). Lin et al. found that CHMP4C expression was higher in cervical cancer tissues, and high CHMP4C expression was associated with lower survival (Lin et al., 2020). Upregulation of CHMP4C in C-33A cells accelerates cell proliferation, migration, and invasion, whereas downregulation of CHMP4C in Ca Ski cells had the opposite effect. Chay et al. reveal that increased FOXO1 and PAX3 expression in cervical cancers indicates an oncogenic role of FOXO1 in cervical cancer cells that correlates with poor patient survival. The findings above are consistent with our results. Interestingly, FOXO1 has also been found to act as a tumor suppressor in cervical cancer cells (Prasad et al., 2014; Aishanjiang et al., 2018). This suggests that FOXO1 has a dual role in cervical cancer and needs to be further researched. To sum up, these DE-ARGs are involved in many important cervical cancer-associated biological functions and pathways.

This study still has some limitations. First, the total samples were randomly divided into train set and test set. Therefore, the sample size of each group was relatively small. Secondly, due to a large number of missing data, some clinical variables were not included for analysis. Thirdly, the molecular mechanism of autophagy affecting the prognosis of CC patients and its significance for clinical translational therapy need to be further studied.

## Conclusion

A risk prediction model based on CHMP4C, FOXO1, and RRAGB was successfully constructed, which could effectively predict the prognosis of CC patients. This model can provide a reference for CC patients to make precise treatment strategy.

## Data Availability Statement

Publicly available datasets were analyzed in this study. This data can be found at: https://portal.gdc.cancer.gov/; https://www.ncbi.nlm.nih.gov/geo/query/acc.cgi.

## Author Contributions

HS was responsible for the original draft writing, acquisition, investigation, conceptualization, visualization, and software. FZ, XY, ZS, and FO were involved in the methodology. ZX and YZ were involved in the conception and design of the study and revised the manuscript. All authors have read and agreed to the final version of the manuscript.

## Conflict of Interest

The authors declare that the research was conducted in the absence of any commercial or financial relationships that could be construed as a potential conflict of interest.

## Supplementary Material

The R code of the pipeline is available at https://github.com/qwjdhsoidkjf/2020-12-20.

Supplementary Figure 1 Differentially expressed autophagy-related genes. **(A)** The heat map of differential expression of ARGs. **(B)** Volcano plot of the differential expression of 531 autophagy-related genes in CC samples.

Supplementary Figure 2 **(A)** LASSO coefficient spectrum of 3 DE-ARGs in CC. Generate a coefficient distribution map for a logarithmic (λ) sequence. **(B)** Selecting the best parameters in the LASSO model (λ).

Supplementary Figure 3 **(A)** The Kaplan-Meier curve demonstrates that patients in high-risk group has a poorer prognosis in stage I&II subset. **(B)** Patients in high-risk group has a poorer prognosis in stage III&IV subset. **(C)** Patients in high-risk group has a poorer prognosis in grade 1&2 subset. **(D)** Patients in high-risk group has a poorer prognosis in grade 3&4 subset.

Supplementary Figure 4 **(A)** The Kaplan–Meier curve demonstrates that patients in high-risk group has a lower disease free survival rate in the external validation set (GSE44001). **(B)** Time-dependent ROC curve analysis for disease free survival prediction by the risk score in the external validation set.
