# Supplementary figures and images for: Application of an Autophagy-Related Gene Prognostic Risk Model Based on TCGA Database in Cervical Cancer
Source: Front Genet. 2021 Feb 9;11:616998. doi: 10.3389/fgene.2020.616998 (PMC7900625; doi:10.3389/fgene.2020.616998)

A

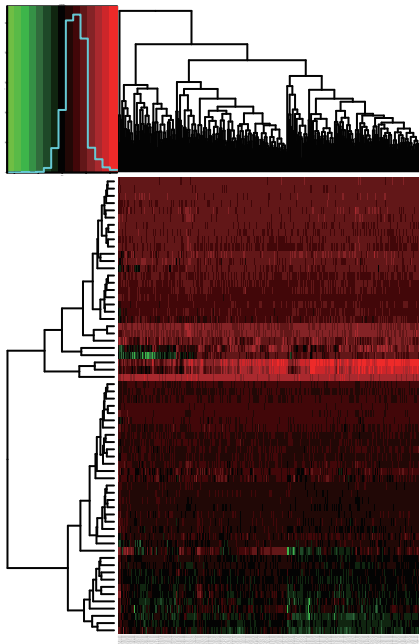

B

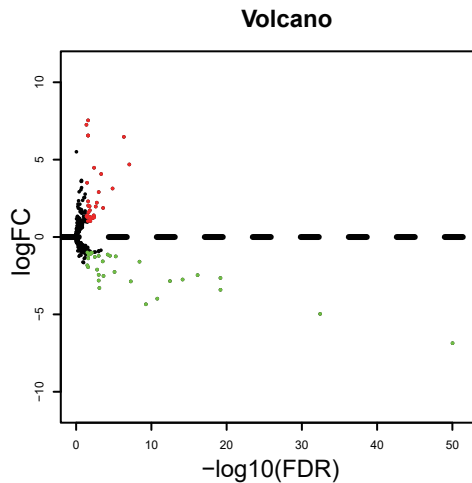

Supplement: Supplementary Figure 1 — Differentially expressed autophagy-related genes. (A) The heat map of differential expression of ARGs. (B) Volcano plot of the differential expression of 531 autophagy-related genes in CC samples. [file Data_Sheet_1.PDF]

A

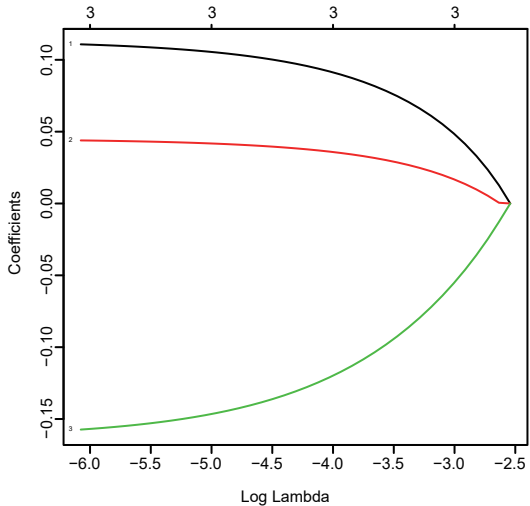

B

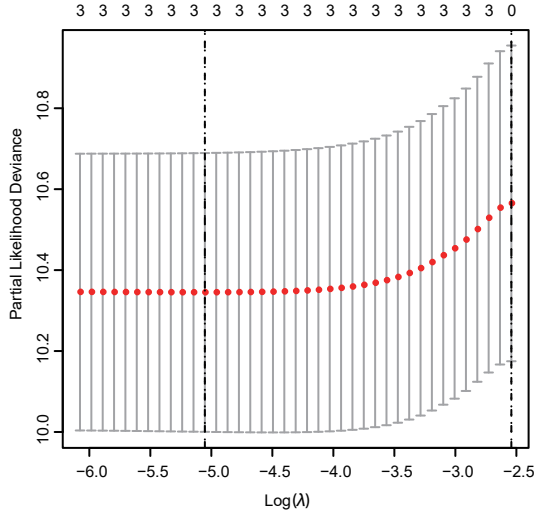

Supplement: Supplementary Figure 2 — (A) LASSO coefficient spectrum of 3 DE-ARGs in CC. Generate a coefficient distribution map for a logarithmic (λ) sequence. (B) Selecting the best parameters in the LASSO model (λ). [file Data_Sheet_2.PDF]

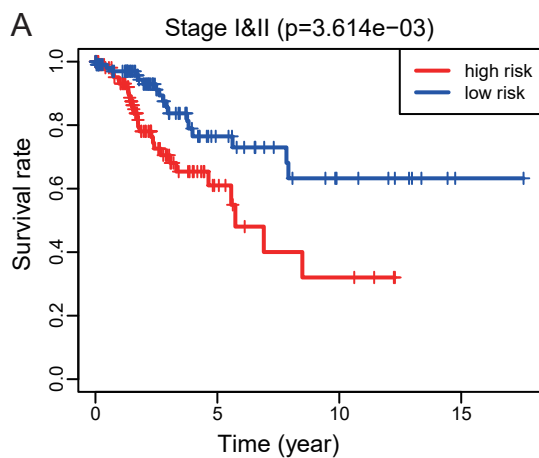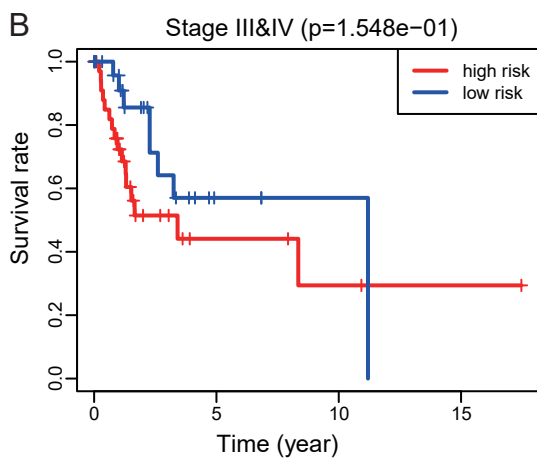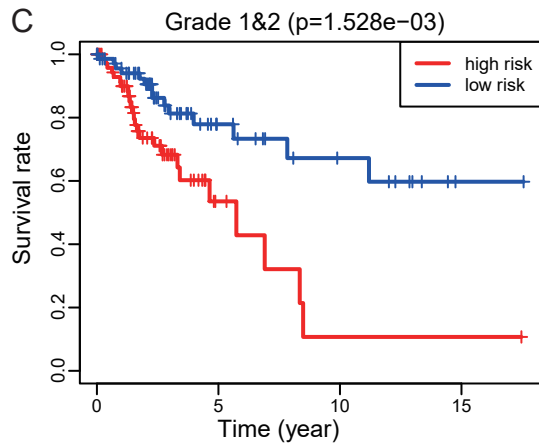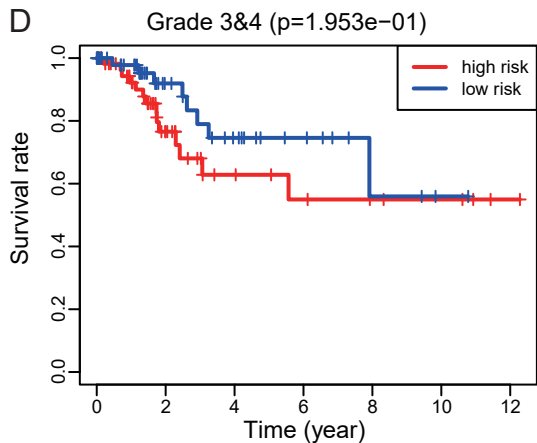

Supplement: Supplementary Figure 3 — (A) The Kaplan-Meier curve demonstrates that patients in high-risk group has a poorer prognosis in stage I&II subset. (B) Patients in high-risk group has a poorer prognosis in stage III&IV subset. (C) Patients in high-risk group has a poorer prognosis in grade 1&2 subset. (D) Patients in high-risk group has a poorer prognosis in grade 3&4 subset. [file Data_Sheet_3.PDF]

A

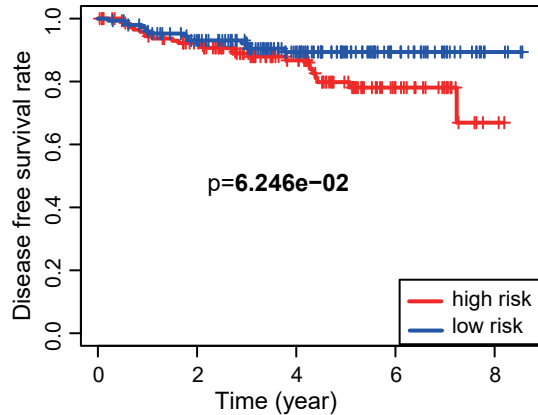

B

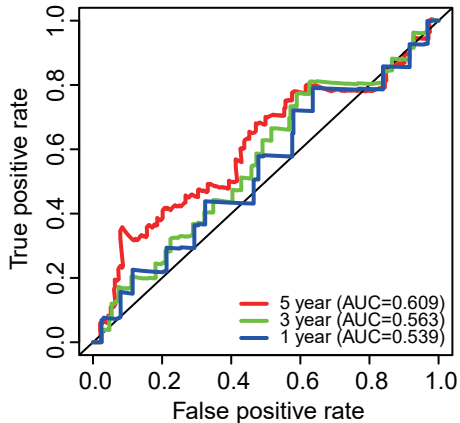

Supplement: Supplementary Figure 4 — (A) The Kaplan–Meier curve demonstrates that patients in high-risk group has a lower disease free survival rate in the external validation set (GSE44001). (B) Time-dependent ROC curve analysis for disease free survival prediction by the risk score in the external validation set. [file Data_Sheet_4.PDF]
